# Assessing the Performance of Different Treatment Methods in Removing Tetracycline from Wastewater: Efficiency and Cost Evaluation

**DOI:** 10.3390/ma18092134

**Published:** 2025-05-06

**Authors:** Kehinde Shola Obayomi, Zongli Xie, Stephen R. Gray, Jianhua Zhang

**Affiliations:** 1 Institute for Sustainable Industries and Liveable Cities, Victoria University, Werribee, VIC 3030, Australia; stephen.gray@vu.edu.au; 2 Zuckerberg Institute for Water Research, The Jacob Blaustein Institutes for Desert Research, Ben-Gurion University of the Negev, Sde-Boker Campus, Midreshet Ben-Gurion 8499000, Israel; 3 Department of Chemical Engineering, Curtin University, CDT 250, Miri 98009, Malaysia; 4 Commonwealth Scientific and Industrial Research Organization (CSIRO) Manufacturing, Private Bag 10, Clayton South, VIC 3169, Australia; zongli.xie@csiro.au

**Keywords:** adsorption, photocatalytic degradation, ozonation, tetracycline, cost analysis

## Abstract

To tackle the pollution of tetracycline (TC) in aqueous environments, a few treatment methods, including ozonation, adsorption, and photocatalytic degradation, were compared using a novel and sustainable granular activated carbon-based zinc oxide nanoparticle (ZnO@GAC) composite. The results demonstrate that the ZnO@GAC composite towards TC exhibited a high removal efficiency of 82.1% in a batch adsorption system. Moreover, the photocatalytic TC degradation study on ZnO@GAC under UV light yields a maximum degradation efficiency of 86.4% with a pseudo-first-order rate constant value of 0.0059 min^−1^. Ozonation treatment resulted in TC and total organic carbon (TOC) removal reaching a maximum of 95.3% and 79.7% for 4 mg O_3_/min and 99.6% and 86.6% for 16 mg O_3_/min after 10 min. Overall, in comparing the adsorption, photocatalysis, and ozonation techniques, in terms of removal efficiency and time, ozonation was found to be more promising for treating TC, while in terms of cost-effectiveness, the adsorption process is preferable. Finally, the application of the developed composite in municipal and hospital wastewater using adsorption, photocatalytic degradation, and ozonation techniques revealed that the TOC removal efficiencies were higher for hospital wastewater than municipal wastewater. Furthermore, the applicability of these techniques in treating hospital wastewater containing pharmaceuticals, antibiotics, fungicides, and antimicrobial pollutants shows an outstanding result after treatment. In conclusion, the technologies studied in this research can significantly improve the efficiency and effectiveness of wastewater treatment applications, providing a sustainable, cost-effective, and eco-friendly solution.

## 1. Introduction

The extensive use of antibiotics results in increased antibiotic-containing wastewater being generated and released into the environment, raising serious environmental concern [1]. Siahbandi et al. [2] reported in a recent study that the amount of antibiotics that entered the environment was about 30–90% and that the residue concentrations of pharmaceutical and hospital wastewater reached between 100 and 500 mg/L. Moreover, the excessive release of antibiotics into the ecosystem can pose a severe threat to human health, including mutagenic, carcinogenic, toxic, and allergic effects due to their chemical stability and biological activity in the environment [3]. Tetracycline (TC), which is one of the most commonly used antibiotics, has gained popularity worldwide for its ability to effectively treat and prevent bacterial infections in humans and animals, and enhance the growth of animals and livestock owing to its broad bacteriostatic spectrum and good antibacterial activity [4,5]. However, based on previous studies, about 60–80% of unmetabolized TC is discharged in the form of microbial and chemical effluents into the environment through human and animal excretion, posing a severe threat and irreparable damage to the ecosystem [6,7]. Therefore, it is necessary and significant to develop effective, environmentally friendly, cost-effective, and sustainable techniques for TC-containing wastewater to remove TC from the environment.

Several treatment techniques, including filtration, coagulation, electrochemical treatment, the Fenton-like method, reverse osmosis, photocatalytic degradation, and adsorption, have been well documented for the removal of TC from wastewater [4,8,9,10]. However, most of these techniques have drawbacks such as high cost, high energy consumption, sludge formation, and secondary pollution formation [11,12]. In recent years, the integration of adsorption and photocatalysis has emerged as a promising technique for organic pollutant remediation, likely due to its high removal efficiency, recyclability, long-term stability, simplicity, and environmental friendliness [13]. Specifically, organic pollutants with low concentrations are firstly adsorbed by adsorbents and then degraded in situ through photocatalysis. Suffice to state that the innovative strategy can be employed to solve the challenges of adsorbent saturation and photocatalytic separation [14]. Furthermore, the integration of photocatalytic and absorptive technologies is becoming increasingly intractable for the treatment of pollutants, with the robust advantage of averting mass transfer resistance by pre-adsorption and achieving in situ photodegradation via photocatalysis under visible light [15]. In this regard, the development of a novel, cost-effective, and sustainable adsorbent with a dual function of adsorptive–photocatalytic performance capable of adsorbing and degrading TC effectively in an eco-friendly approach is highly desirable.

In recent years, semiconductors such as titanium oxide [16], cadmium sulfide [17], and zinc oxide [18] have been employed as photocatalysts in environmental applications due to their excellent optical properties, photocatalytic activity under visible light irradiation, and degradation efficiency [6]. Among these, ZnO has attracted considerable attention as a promising and desirable photocatalyst in the field of photocatalytic degradation due to its suitable band gap (3.37 eV) and high photosensitivity. ZnO has properties such as a high oxidation state, excellent photocorrosion resistance, good chemical stability, non-toxicity, controllable morphological structure, and high excitation energy (0.06 eV), making it an excellent photocatalyst [19]. However, the photocatalytic performance of ZnO in practical applications is still unsatisfactory due to poor light utilization, positive conduction band-limited pollutant adsorption on semiconductors, low transfer efficiency, and fast recombination of photo-induced charge carriers [14,20]. Furthermore, due to their photosensitization effect, ZnO agglomerates easily and becomes unstable during photocatalytic activity [18]. To overcome these drawbacks, incorporating ZnO into porous materials such as carbon-based materials was proposed as an effective strategy to enhance their stability, photo-generated electron migration rate, and adsorptive–photocatalytic synergy performance. Moreover, the in situ adsorption of ZnO on a carbonaceous material will not only promote the adsorption of TC but will also effectively improve the separation of photo-excited electron-holes, expand the absorption band range to the visible light spectrum, facilitate mass diffusion, and enhance light transmittance [21].

Activated carbon (AC), a type of carbonaceous material, has proven to be highly effective as an adsorbent in environmental remediation applications [22]. Activated carbon has been widely employed as one of the most effective adsorbents in wastewater treatment due to its high surface area, low cost, excellent adsorptive performance with good surface chemistry, and well-developed porous structure combinations [23]. Due to its high chemical stability and surface area, the use of activated carbon as a support could help reduce the photogenerated electron transfer and agglomeration of ZnO particles to enhance the photodegradation performance [24]. For example, Li et al. [25] recently reported that the photodegradation performance was significantly enhanced after impregnation with a carbonaceous material via petrochemical enrichment at the photocatalyst interface, thereby mediating oxidative degradation reactions through a non-radical mechanism [26]. While several studies have reported the extensive use of ZnO semiconductors and activated carbon as photocatalyst and adsorbent for TC degradation and adsorption, to the best of our knowledge, no study has reported the dual function of ZnO and granular activated carbon composites (ZnO@GAC) with synergistic adsorptive–photocatalytic capability towards TC.

Furthermore, the photocatalytic degradation mechanism of TC under UV light involves three stages: 1] adsorption of TC on the photocatalyst surface; 2] subsequent excitation of adsorbed AC by UV light, resulting in the injection of electrons into the photocatalyst conduction band to generate conduction band electrons (eCB−), which are then scavenged by O_2_ to produce hydroxyl radicals (OH.); and 3] the produced hydroxyl radicals being responsible for TC degradation into a non-toxic product. Thus, among these stages, TC adsorption onto the photocatalyst surface is crucial for effective degradation under UV light [27].

Motivated by these findings from previous studies, this study aimed to design a composite material through the fabrication of zinc oxide-loaded granular activated carbon (GAC) through the in situ hydrothermal method with an enhanced active site for TC removal from an aqueous system using different techniques. Furthermore, several studies have employed different removal techniques for TC removal, owing to its frequent presence in aqueous media. In addition, understanding the treatment and utility costs associated with these various treatment methods is of significant importance. However, previous studies have reported the treatment costs (operational and investment) for individual techniques; only a few have conducted comparative analyses, due to the inherent challenges in such comparisons and the variability in underlying factors. This study also attempted to address this gap by performing comparative experiments to examine TC removal rate, degradation efficiencies, and cost-effectiveness across different treatment methods, including ozonation, photocatalysis, and adsorption. Finally, this study provides insight into the practical applicability of ZnO@GAC for eliminating TC in complex wastewater.

## 2. Materials and Method

### 2.1. Materials

Analytical-grade chemicals and materials, including tetracycline (TC), zinc acetate dihydrate (Zn(CH_3_COO)_2_·2H_2_O), and granular activated carbon (GAC), were sourced from Merck Chemical, Australia, and used without further purification. The synthesis method described by Obayomi et al., [28] was employed in this study. Briefly, 21.95 g of Zn(CH_3_COO)_2_·2H_2_O was put into a 250 mL beaker containing 100 mL of double distilled water (DDW). The mixture was then placed on a magnetic stirrer with a shaking at 200 rpm at a controlled room temperature for 15 min to ensure complete dissolution and obtained a clear solution. Next, 5 g of GAC was added to the mixture under continuous stirring for another 2 h. Thereafter, the suspended mixture was centrifuged, washed with DDW 5 times, and dried in a hot-air oven for 12 h at 80 °C. The obtained dried solid was then annealed in air at 400 °C for 2 h. Finally, the resulting black solid named ZnO@GAC was stored in sealed plastic containers for further experiments. The textural properties of ZnO, GAC, and ZnO@GAC are summarized in Table 1, and these were also reported in our previous study [29].

### 2.2. Batch Adsorption Experiment

In a typical batch adsorption experiment, 50 mg of ZnO@GAC and GAC were transferred into a set of 250 mL beakers containing 100 mL of TC solution (50 mg/L) while maintaining the initial pH solution (pH 5.5). The beaker containing the mixture was stirred on a magnetic stirrer at a controlled room temperature with a constant stirring speed of 160 rpm for a predetermined time. Prior to the attainment of equilibrium, TC samples were withdrawn at different time intervals, and filtered through a 0.45 μm filter membrane, and the residual TC concentrations and total organic carbon (TOC) were measured with the aid of a UV-VIS spectrophotometer (DR5000 Hach spectrometer, Dandenong South, Australia) at a maximum wavelength of 365 nm and a TOC-VCSH analyzer (Shimadzu, Kyoto, Japan), respectively. The TC removal efficiency and uptake capacity, q_t_ at time t, were computed using the following equations:

(1) $$\%\;TC\;removal=\left(\frac{C_{0}-C_{t}}{C_{o}}\right)\times 100$$

(2) $$q_{t}=\frac{\left(C_{0}-C_{t}\right)\;V}{W}$$

where $C_{o}$ and $C_{t}$ are the initial and final TC concentrations (mg/L), W is the catalyst weight (g), and V is the solution volume of TC.

### 2.3. Photocatalytic Degradation Experiment

The photocatalytic degradation activity of ZnO@GAC towards TC under UV light was investigated in a batch system using a photocatalytic reactor. Specifically, 50 mg of ZnO@GAC and GAC were measured and added to a 250 mL beaker containing 100 mL of TC solution (50 mg/L), and the initial pH of the solution was maintained throughout the experiment. Thereafter, the beaker containing the mixture was stirred with a speed of 160 rpm at controlled room temperature (298 K) for 30 min in the dark prior to irradiation to establish catalyst adsorption–desorption equilibrium. Next, the photocatalytic reactor UV-lamps (UV-A irradiation) were turned on to initiate the photocatalytic reaction, and the suspension was subjected to irradiation for 7 h. The distance between the light and the beaker was about 20 cm, with irradiance of 2–2.5 mW/cm^2^. Next, TC samples were withdrawn at defined time intervals and filtered through a 0.45 μm filter, and the residual TC concentrations were measured with the aid of a UV-VIS spectrophotometer (DR5000 Hach spectrometer, Australia) at a maximum wavelength of 365 nm. TOC was measured using a TOC-VCSH analyzer (Shimadzu, Japan). The TC photocatalytic degradation efficiency was calculated using UV-Vis/TOC:

(3) $$TC\;degradation(\%)=\left(\frac{C_{0}-C_{t}}{C_{o}}\right)\times 100$$

(4) $$\mathrm{TOC}\;\mathrm{removal}\;(\%)=\frac{{TOC}_{0}-{TOC}_{t}}{{TOC}_{0}}\times 100$$

where ${TOC}_{0}$ and ${TOC}_{t}$ are the initial TOC concentrations and those at a time t.

### 2.4. Ozonation Experiment

The ozonation experiment for TC treatment was conducted in a column-type reactor with a capacity of 1 L. An ozone generator produced 50 mg/min of ozone when fed pure oxygen at a flow rate of 4 L/min, which was subsequently bubbled through water using a glass tube with a sintered end. The experiment was conducted in a 1000 mL glass beaker containing 500 mL of TC solution (50 mg/L) while maintaining the initial pH of the TC solution at a controlled room temperature. Thereafter, ozone was continuously bubbled at different ozone concentrations (4 mg-O_3_/min and 16 mg-O_3_/min). During the ozonation process, TC solution was sampled at different intervals of time (0, 1, 3, 5, 7, and 10 min) and measured to determine the residual TC concentrations with the aid of a UV-VIS/TOC using the equations presented in (3) and (4), respectively.

### 2.5. Municipal and Hospital Real Wastewater Treatment

The applicability of ZnO@GAC in treating a municipal and hospital wastewater collected from a collected wastewater treatment plant and hospital in South Australia was examined. The effect of inhomogeneous particulate impurities in the solution was eliminated by filtering the wastewater with a 90 mm Whatman filter paper. Thereafter, 50 mg of the prepared ZnO@GAC was transferred into a 250 mL beaker containing 100 mL of the wastewater solution. The wastewater treatment experiment was conducted using the adsorption, photocatalytic degradation, and ozonation methods under equilibrium conditions.

## 3. Results and Discussion

### 3.1. Adsorptive Performance

**Figure 1:**
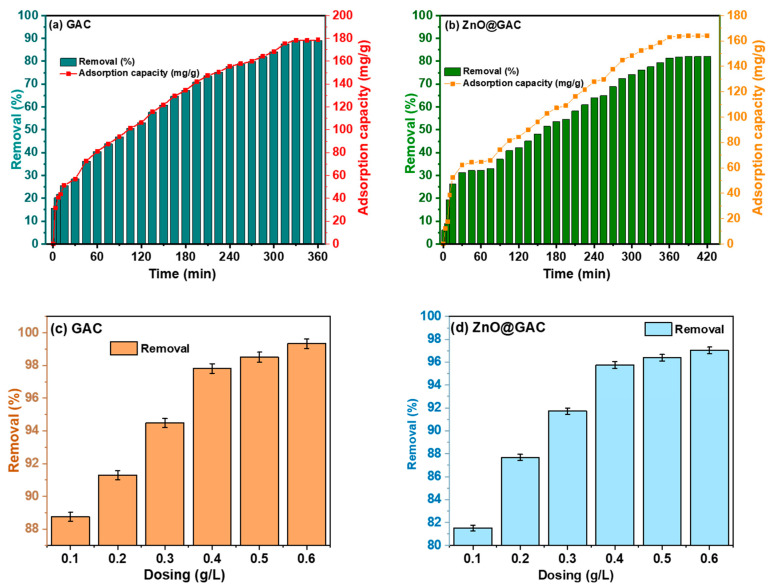
Effect of contact time (**a**,**b**) and adsorbent dosage (**c**,**d**) on TC adsorption towards GAC and ZnO@GAC (C_O_ = 50 mg/L, adsorbent dosage = 50 mg, volume of solution = 100 mL, temperature = 298 K, and pH = 5.5).

The adsorptive performance of GAC and ZnO@GAC nanocomposites towards TC adsorption was investigated at a catalyst dosage of 50 mg and an initial TC concentration of 50 mg/L while maintaining the TC initial pH. As presented in Figure 1a,b, the TC removal efficiency and adsorption capacity towards GAC and ZnO@GAC increased with increasing contact time. The results demonstrate that the adsorption removal efficiency and adsorption capacity of TC towards GAC were 87.7% and 178.6 mg/g, outperforming those of ZnO@GAC, which were observed to be 82.1% and 164.1 mg/g, respectively. The prevailing performance of GAC could be attributed to the abundance of adsorption sites in the form of hydroxyl groups and the larger surface area responsible for binding the TC to the GAC surface, facilitating the adsorption of TC [30]. On the other hand, the adsorption site blockage resulting from the incorporation of ZnO on the GAC surface could have reduced the TC removal efficiency and adsorption capacity [31]. Also, it was observed that TC adsorption was rapid at the initial stage but gradually became slower until dynamic equilibrium was established. Furthermore, the TC adsorption towards GAC attained sorption equilibrium within 330 min, while that of ZnO@GAC reached sorption equilibrium at a longer time (390 min), a point where the amount of TC adsorbed on GAC and ZnO@GAC is in a state of dynamic equilibrium with the TC amount desorbing from the adsorbents.

The effect of GAC and ZnO@GAC dosing on TC adsorption was further examined by varying the GAC and ZnO@GAC dosing concentrations from 0.1 to 0.6 g/L while maintaining the initial TC concentration (50 mg/L) and pH (5.5) at an equilibrium time of 330 and 390 min, respectively. The result depicted in Figure 1c,d reveals that the TC removal efficiency and adsorption capacities increased by gradually increasing the GAC and ZnO@GAC dosage concentrations. The increase in TC removal and adsorption capacity with increasing dosage concentration could be attributed to the availability of more adsorption sites at higher dosages, which promotes the adsorption of more TC [32]. Furthermore, the TC removal efficiency and adsorption capacity of GAC increase from 88.7% to 99.3%, while that of ZnO@GAC increases from 81.5% to 97.02%, respectively.

### 3.2. Photocatalytic Performance

**Figure 2:**
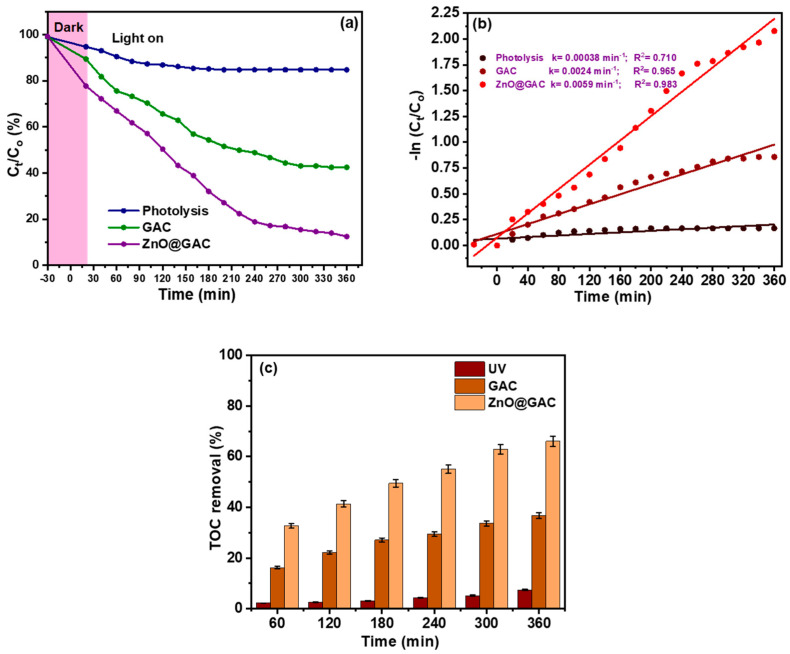
The plots of (**a**) photocatalytic performance of GAC and ZnO@GAC towards TC degradation, (**b**) pseudo-first-order kinetics, and (**c**) TOC removal rate of TC degradation under UV light (C_0_ = 50 mg/L, volume of solution = 100 mL, catalyst dosage = 50 mg, pH = 5.5, and temperature = 298 K).

Figure 2 shows the photocatalytic degradation performance of GAC and ZnO@GAC on TC. First, the degradation performance of GAC and ZnO@GAC towards TC was subjected to a dark reaction for 30 min to establish equilibrium adsorption–desorption as presented in Figure 2a. The photocatalytic degradation of TC under UV light only was observed from the degradation curve not to have degraded on its own, indicating that the TC is photo-stable [33]. However, the TC degradation in the presence of GAC and ZnO@GAC under UV light illumination was 52.5% and 86.4%, respectively, after 360 min. The outstanding photocatalytic performance of ZnO@GAC towards TC degradation could be attributed to the availability of more active sites and the presence of effective charge transfer, which is able to support the localization of oxygen vacancies and anatase-rutile junction structure as well as inhibit charge carrier recombination [34,35]. In addition, the modification of GAC with ZnO expanded the range of UV light adsorption and enhanced the photoinduced transfer carrier, thus promoting the photocatalytic degradation performance of ZnO@GAC [36]. Furthermore, the experimental data of the photocatalytic degradation of TC using GAC and ZnO@GAC were fitted to the pseudo-first-order kinetics equation given by

(5) $$\mathrm{Pseudo}\text{-}\mathrm{first}\;\mathrm{order}\text{:}-\ln\frac{C_{t}}{C_{o}\;}=kt$$

The TC photocatalytic degradation kinetics curve displayed in Figure 2b shows a good linear relationship with time, suggesting that the TC degradation on GAC and ZnO@GAC could be best explained by the pseudo-first-order model [37]. The photocatalytic degradation rate kinetics constants of TC under UV light (without catalyst), GAC, and ZnO@GAC were 0.00038, 0.0024, and 0.0059, respectively. Notably, the rate kinetic constant of TC degradation onto ZnO@GAC was observed to be 15.5 and 2.5 times that of UV (without catalyst) and GAC, respectively, demonstrating that the ZnO@GAC exhibited a superior degradation performance. Furthermore, the TOC removal rate by GAC and ZnO@GAC after photodegradation, as shown in Figure 2c, revealed that within 60 min, only 16.2% and 32.7% mineralized. However, after 360 min of degradation time, the TOC removal rate of TC by UV-light only, GAC, and ZnO@GAC was observed to be 7.4%, 36.8%, and 66.1.%, respectively. The partial mineralization of TC by UV-light only, GAC, and ZnO@GAC could be attributed to the partial TC oxidation and its immediate photodegradation by reactive species [38]. Furthermore, the TOC removal rate increase with increasing irradiation time could be attributed to the fact that incident light irradiation becomes easier as the irradiation increases to penetrate the internal TC solution, thereby promoting the number of photogenerated carriers [39].

### 3.3. Ozonation Performance

**Figure 3:**
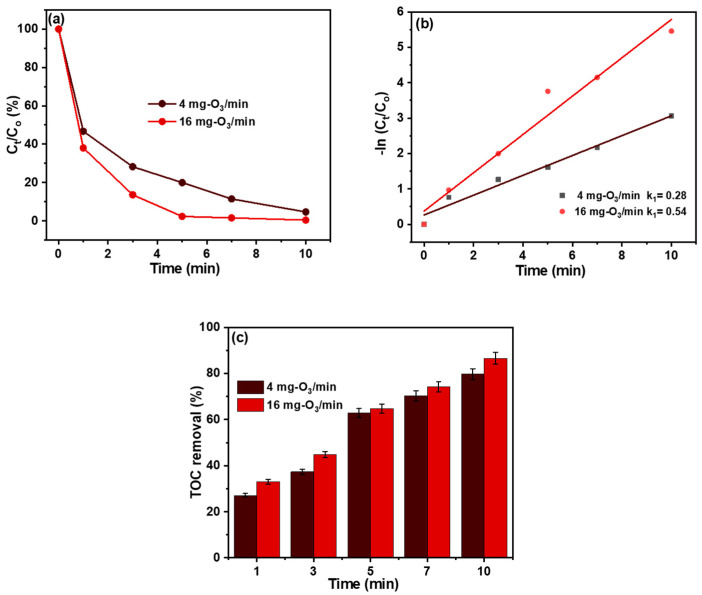
The plots of (**a**) TC ozonation as a function of time, (**b**) pseudo-first-order kinetics, and (**c**) TOC removal rate of TC under the influence of ozonation (C_o_ = 50 mg/L, pH = 5.5, temperature = 298 K, and volume of solution = 100 mL).

The TC degradation under the influence of ozonation and the determination of their performance at different dosing concentrations (4 mg-O_3_/min and 16 mg-O_3_/min) were investigated. As shown in Figure 3a, with an increase in the ozonation dosage from 4 mg-O_3_/min to 16 mg-O_3_/min, the TC removal rate after 10 min increased from 95.3% to 99.6%. The increase in the TC degradation rate with increasing ozone concentration could be attributed to the fact that at increased dosing concentrations, the mass transfer driving force is enhanced, thereby promoting the ozone volumetric mass transfer coefficient from the gaseous phase to the liquid phase [40]. Moreover, the pseudo-first-order kinetics plot depicted in Figure 3b demonstrates that the reaction rate constant (k_1_) of 16 mg-O_3_/min (0.58 min^−1^) was observed to be about 2.1 times higher than that of 4 mg-O_3_/min (0.28 min^−1^), suggesting that the increase in dosing concentration promotes TC ozonation [41]. Furthermore, the TC mineralization presented in Figure 3c reveals that the TOC removal efficiency increased with increasing dosing concentration and ozonation time, from 27.1% (1 min) to 79.7% (10 min) for 4 mg-O_3_/min and 33.04% (1 min) to 86.6% (10 min) for 16 mg-O_3_/min, respectively. This suggests that only 20.3% and 13.4% of degraded TC cannot be completely mineralized and transformed into carbon dioxide; therefore, higher TC mineralization leads to TC toxicity reduction [42]. Furthermore, this demonstrates that at higher dosing concentrations, the ozone (O_3_) generated sufficient ability to destroy and transform almost all the dissolved organic content in TC into carbon dioxide (CO_2_), thereby reducing their toxicity [43] Thus, if higher TC is to be removed, then a higher dosing concentration may be needed to achieve it.

### 3.4. Comparative Study of Different Techniques in Real-Wastewater Treatment

**Figure 4:**
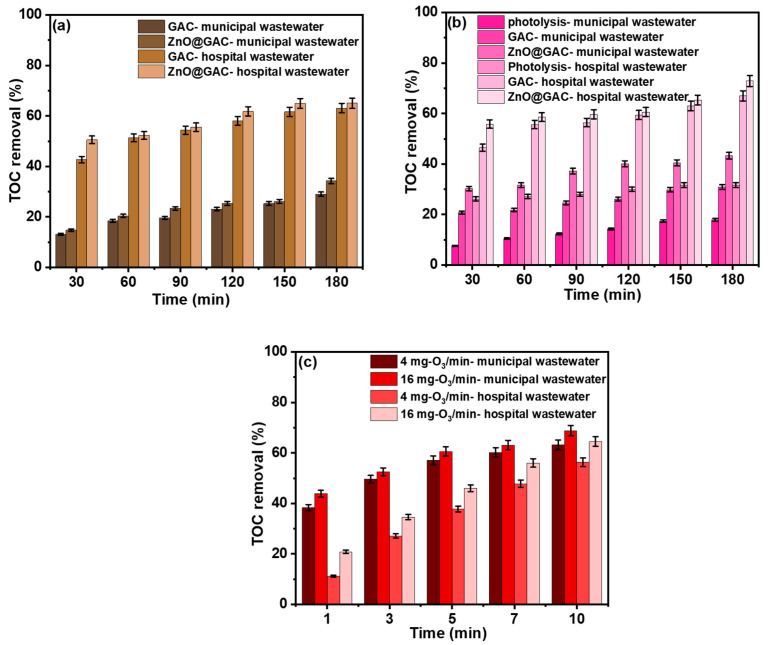
TOC removal during (**a**) adsorption (**b**) photocatalytic degradation, and (**c**) ozonation of municipal and hospital wastewater (volume of solution = 100 mL and catalyst dosage = 50 mg).

The performance of the adsorption, photodegradation, and ozonation techniques was investigated in municipal and hospital wastewater. The results are depicted in Figure 4. All techniques revealed an increase in the TOC removal rate with increasing time. This could be attributed to the fact that the breakdown component concentration, which has the capacity to convert the organic compounds in municipal and hospital wastewater, may be responsible for this observation and could be associated with a longer time [44]. Firstly, as shown in Figure 4a, it was observed that TOC removal using the adsorption technique for municipal wastewater treatment by GAC and ZnO@GAC was 29.0% and 34.3%, while that of hospital wastewater on GAC and ZnO@GAC was 63.1% and 65.0% within 180 min. These results indicate that about 71.0% and 65.7% of municipal wastewater and 36.9% and 35.0% of hospital wastewater were not completely mineralized [45]. Secondly, the photocatalytic performance of GAC and ZnO@GAC in degrading municipal and hospital wastewater under the influence of UV light within 180 min is also presented in Figure 4b. The results show that the TOC removal efficiency using photocatalysis (UV light only), GAC, and ZnO@GAC to degrade municipal wastewater was 17.9%, 30.9%, and 67.0%, while the TOC removal rate for hospital wastewater was 21.7%, 43.3%, and 72.9%, suggesting that ZnO@GAC is a better purifying agent and exhibits a good oxidative degradation performance towards municipal and hospital wastewater [46]. Thirdly, the TOC removal efficiency recorded using the ozonation technique at different dosing concentrations (4 mg O_3_/min and 16 mg O_3_/min) for municipal wastewater was 63.2% and 68.8%, while that of hospital wastewater was 56.3% and 64.5% within the 10 min ozonation time (Figure 4c). These results demonstrate that the ozonation process could degrade municipal and hospital wastewater into various refractory oxidative intermediates but completely into H_2_O and CO_2_ [47]. In addition, the results suggest that better TOC removal efficiencies for municipal and hospital wastewater were achieved with an ozone concentration of 16 mg/L compared to 4 mg/L. However, both 4 mg O_3_/min and 16 mg O_3_/min still provide satisfactory TC removal efficiency.

Furthermore, the different methods studied were also tested in hospital wastewater containing different pollutants, including antibiotics, fungicides, antimicrobials, and pharmaceuticals, and their removal efficiencies are depicted in Table 2. The results show that the different methods show an outstanding performance in treating hospital wastewater. However, the use of UV light alone shows low-percentage removal towards propylparaben.

### 3.5. Cost Analysis

A cost analysis study is one of the important factors that must be considered before the selection of any wastewater treatment techniques. Thus, this study tries to understand the economic impact of the various treatment techniques, including adsorption, photocatalytic degradation, and ozonation, that were employed for TC treatment in aqueous media. However, the ozonation technique is an electrical-energy-intensive consumption process, and the electrical energy can represent a major part of the operational costs. Therefore, the electrical energy per order of TC removal (EE/O) was employed and is given by the equation

(6) $$\mathrm{EE}/O=\frac{P_{elect}\times t\times 1000}{v\times 60\times k_{1}}$$

where $P_{elect}$ is the ozonation system’s rated power (0.15 kW for ozone generator); t is the ozonation reaction time (min); v is the volume of TC (L); and $k_{1}$ is the pseudo-first-order rate constant (min^−1^). The total cost of producing ZnO@GAC with the inclusion of material, chemical, electricity, and overhead cost is depicted in Table 3. The result in the table reveals that the estimated cost of synthesizing 20.34 g of ZnO@GAC is only USD 5.59.

In comparison, the results presented in Table 3 reveal that the cost of treating 100 L of TC containing wastewater using the adsorption technique is only USD 154.0, which is cheaper than photodegradation (USD 186.80) and ozonation (USD 6789 and USD 3518). Although, the ozonation process outperformed the adsorption and photodegradation techniques in terms of their removal efficiency, considering the economical, sustainability, environmental, and regenerability benefits, the adsorption technique was more suitable and promising in removing TC from wastewater.

## 4. Conclusions

In conclusion, this research study aimed to develop a sustainable and environmentally friendly dual-function ZnO@GAC material with adsorptive and photocatalyst performance via an in situ hydrothermal method to treat TC in an aqueous environment. Moreover, the removal efficiencies and cost-effectiveness were compared using different treatment techniques such as adsorption, photocatalytic degradation, and ozonation in municipal and hospital wastewater. The adsorptive performance and adsorption capacity of GAC towards TC was 87.7% and 178.6 mg/g, outperforming that of ZnO@GAC, which was observed to be 82.1% and 164.1 mg/g. Under UV light, the ZnO@GAC photocatalyst demonstrated outstanding photocatalytic performance, achieving 86.4% degradation of TC within 360 min compared to GAC’s degradation efficiency of 52.5%. The degradation performance of ZnO@GAC towards TC followed pseudo-first-order kinetics, and the rate constant is 2.5 times higher than that of GAC. The superiority of the ZnO@GAC composite in TC photocatalytic degradation could be attributed to its unique morphology, which enhances its light absorption ability and effectively prevents charge carrier recombination. Notably, the TOC removal rates of TC by GAC and ZnO@GAC were 36.8% and 66.1.3%, respectively. Furthermore, under the influence of ozonation, the TC was almost completely degraded after 10 min. With an increasing ozonation dosage from 4 mg/min to 16 mg/min, the TC removal rate increased from 95.3% to 99.6%. The TOC removal efficiency after 10 min was 79.7% and 86.6% for 4 mg O_3_/min and 16 mg O_3_/min, respectively, demonstrating that only 20.3% and 13.4% of degraded TC cannot be totally mineralized and transformed to carbon dioxide and may generate intermediates; therefore, higher TC mineralization leads to TC toxicity reduction. In comparison, the mineralization of organic pollutants in municipal and hospital wastewater using adsorption, photocatalytic degradation, and ozonation revealed higher removal efficiency for hospital wastewater than municipal wastewater, which could be attributed to the wastewater characteristics. Finally, in terms of cost, the adsorption process is assumed to be more economical than the other techniques following the order adsorption > photocatalysis > ozonation.

## Figures and Tables

**Table 1:** Characteristics of GAC and ZnO@GAC.

Materials	BET Surface Area (m^2^/g)	Total Pore Volume (cm^3^/g)	Pore Diameter (nm)
ZnO	74	0.00234	2.87
GAC	474	0.268	2.26
ZnO@GAC	453	0.273	2.42

**Table 2:** Antibiotic removal from hospital wastewater using different treatment methods.

Treatment Methods	Pollutants/Removal (%)					
	Trimethoprim	Metronidazole	Sulfamethoxazole	Tebuconazole	Propylparaben	Carbamazepine
Wastewater + UV light only	<LOD	<LOD	83	<LOD	14	<LOD
GAC + UV light + wastewater	<LOD	<LOD	<LOD	<LOD	90	<LOD
GAC@ZnO + UV light + wasterwater	<LOD	<LOD	<LOD	<LOD	<LOD	<LOD
GAC + wastewater (adsorption)	<LOD	<LOD	<LOD	<LOD	<LOD	<LOD
GAC@ZnO + wastewater (adsorption)	<LOD	<LOD	<LOD	<LOD	<LOD	<LOD
Wasterwater + ozone (4 mg.O_3_/min)	<LOD	<LOD	<LOD	<LOD	<LOD	<LOD
Wasterwater + ozone (16 mg.O_3_/min)	<LOD	<LOD	<LOD	<LOD	<LOD	<LOD

<LOD (below limit of detection).

**Table 3:** Cost analysis study for TC removal using various techniques.

	Techniques	Cost Description	Unit Cost (USD)	Quantity Used	Total Cost (USD)
1.	Adsorption	ZnO@GAC	5.59/20.34 g	0.5 g	0.14
		Cost of electricity (magnetic stirrer)	0.19/1 kWh	550 W × 6	0.63
	Total cost for adsorbing 0.5 L of TC (50 mg/L)	-	-	-	0.77
	Total amount needed to adsorb 100 L of TC (50 mg/L)				154.0
2.	Photocatalytic degradation	ZnO@GAC	5.59/20.34 g	0.5 g	0.14
		Cost of electricity (magnetic stirrer)	0.19/1 kWh	550 W × 7	0.73
		Cost of electricity (light bulbs)	0.19/1 kWh	(8 W × 6) × 7	0.064
	Total cost for degrading 0.5 L of TC (50 mg/L)	-	-	-	0.93
	Total amount needed to degrade 100 L of TC (50 mg/L)	-	-	-	186.80
3	Ozonation	Energy consumption per unit of TC (EE/O) at 4 mg-O_3_/min	0.19/1 kWh	178.57 kWh	33.93
		Energy consumption per unit of TC (EE/O) at 16 mg-O_3_/min	0.19/1 kWh	178.57 kWh	17.59
	Total cost for ozonating 0.5 L of TC (50 mg/L) at 4 mg-O_3_/min	-	-	-	33.93
	Total amount needed to ozonate 100 L of TC (50mg/L) at 4 mg-O_3_/min				6789
	Total cost for treating 0.5 L of TC (50 mg/L) at 16 mg-O_3_/min	-	-	-	17.59
	Total amount needed to ozonate 100 L of TC (50 mg/L) at 16 mg-O_3_/min				3518

## Data Availability

The original contributions presented in this study are included in the article. Further inquiries can be directed to the corresponding authors.
